# Nutritional Status of Women and Child Refugees from Syria — Jordan, April–May 2014

**Published:** 2014-07-25

**Authors:** Oleg O. Bilukha, Douglas Jayasekaran, Ann Burton, Gabriele Faender, James King’ori, Mohammad Amiri, Dorte Jessen, Eva Leidman

**Affiliations:** 1 Division of Global Health Protection, Center for Global Health, CDC; 2 United Nations High Commissioner for Refugees; 3 Medair International; 4 UNICEF; 5 World Food Programme

As a result of civil war, an estimated 2.8 million refugees have fled Syria and reside in neighboring countries, mainly Lebanon, Turkey, Jordan, and Iraq. The largest Syrian refugee camp in the region is Zaatari camp in Jordan, with approximately 79,000 refugees; another estimated 500,000 Syrian refugees live in Jordanian cities, towns, and villages, mostly in the capital (Amman) and in four northern governorates (Irbid, Mafraq, Jarash, and Zarqa). Although all registered refugees in Jordan receive food vouchers from the World Food Programme (WFP) and vulnerable refugees receive cash assistance from the United Nations High Commissioner for Refugees (UNHCR) and nongovernmental organizations, the nutritional status of some refugees might be compromised because of dislocation, lack of income, and limited access to nutritious foods. To assess the nutritional status of Syrian refugees, UNHCR, WFP, the United Nations Children’s Fund (UNICEF), Medair International (a nongovernmental organization), and CDC, in collaboration with the United Nations Population Fund and the World Health Organization (WHO), conducted cross-sectional, population-representative cluster surveys in Zaatari camp and among refugees residing in the host community. The surveys were conducted during April–May 2014 with the principal objective of assessing nutritional status of refugee children aged 6–59 months and nonpregnant women of reproductive age (15–49 years). Preliminary findings indicated a high prevalence of anemia in Zaatari camp among both children and women (48.4% and 44.8%, respectively). Nutrition policies aimed at ensuring optimal child and maternal micronutrient status and addressing the underlying risk factors for anemia are likely to result in improved health outcomes and a reduction in anemia.

Global acute malnutrition in children aged 6–59 months is the principal indicator of nutritional status in humanitarian emergencies. Hemoglobin measurement is the most feasible method for assessing anemia, as a proxy for micronutrient status of the population in emergency field conditions. The cluster sample in Zaatari camp was selected using the UNHCR population estimates of camp districts and blocks, with systematic random selection of households within clusters. A representative cluster sample of refugees residing in the host community in Jordan was selected using lists of registered refugees provided by UNHCR. Six teams of four members each (an interviewer, two anthropometry measurers, and a hemoglobin measurer) received 6 days of training, including a field test. Children were measured using standard anthropometric procedures (1), and nutritional status was classified based on 2006 WHO growth standards (2). Hemoglobin in women and children was measured using HemoCue Hb 301. Anemia was diagnosed according to WHO thresholds* (3). Oral informed consent was obtained before the interviews and hemoglobin testing.

Data collection in Zaatari camp and in the host community lasted 6 and 10 days, respectively. The final samples in Zaatari camp and the host community included 327 and 483 children aged 6–59 months and 314 and 630 nonpregnant women aged 15–49 years, respectively.

Preliminary findings indicated that the prevalence of global acute malnutrition among children was low both in Zaatari and outside the camp: 1.2% and 0.8%, respectively (Table). Mean weight-for-height z-scores in Zaatari and outside the camp were 0.26 and 0.23, above the WHO standard population mean, indicating that Syrian refugee children in Jordan on average were slightly overweight rather than suffering from wasting. The prevalence of chronic malnutrition (stunting) was significantly higher (p<0.05) in Zaatari compared with children outside the camp: 17.0% compared with 9.0%. Anemia prevalence in Zaatari camp in both children and women exceeded 40% (48.4% and 44.8%, respectively), indicating a problem of major public health significance, according to WHO classification (3). Anemia prevalence outside the camp was 26.1% among children and 31.1% among women, significantly lower (p<0.001 for both children and women) than the prevalence in Zaatari camp (Table).

Unlike in many other humanitarian emergencies, preliminary results indicate that global acute malnutrition is relatively low in the Syrian refugee population in Jordan. The low prevalence of global acute malnutrition among refugee children might result, in part, from the ongoing infant and child feeding interventions supported by UNICEF and blanket distribution of food vouchers by WFP. In contrast, the prevalence of anemia suggests a serious public health problem, especially in Zaatari camp. A nutrition survey conducted in 2013 among Syrian refugees residing in Lebanon reported slightly lower prevalences of anemia compared with the prevalences observed in this survey among refugees residing in the host community: 21.0% compared with 26.1% among children aged 6–59 months and 26.1% compared with 31.1% among women aged 15–49 years, respectively (4).

Nutrition policies aimed at ensuring optimal child and maternal micronutrient status and addressing the underlying risk factors for anemia, especially among refugees in camps, are likely to result in improved health outcomes and a reduction in anemia. Jordan has an existing micronutrient fortification program, supplying the fortified flour for the bread that is provided to refugees in the camp and available for purchase by refugees in the host community. Therefore, one option is to focus on supporting the national fortification program to ensure that refugees have full access to fortified flour products and sustained access to public health programs directed at improving sanitation and hygiene and reducing the risk for morbidity, which might contribute to improving nutritional status.

## Figures and Tables

**TABLE:** Prevalence of global acute malnutrition, stunting, and anemia among Syrian women and child refugees residing in Zaatari camp and outside the camp in the host community — Jordan, April–May 2014

	In Zaatari camp		Outside the camp in the host community	
		
Nutrition standard	%	(95% CI)	%	(95% CI)
**Children aged 6–59 mos**				
Global acute malnutrition*				
Total (WHZ <-2 or bilateral pitting edema)	1.2	(0.5–3.2)	0.8	(0.3–2.2)
Moderate (WHZ -3 to <-2)	0.9	(0.3–2.9)	0.8	(0.3–2.2)
Severe (WHZ <-3 or bilateral pitting edema)	0.3	(0.0–2.4)	0	—
Stunting†				
Total (HAZ <-2)	17.0	(11.7–24.0)§	9.0	(6.5–12.3)§
Moderate (HAZ -3 to <-2)	14.1	(9.6–20.3)	8.1	(5.9–11.2)
Severe (HAZ <-3)	2.9	(1.4–5.8)	0.9	(0.3–2.2)
Anemia¶				
Any anemia (Hb <11 g/dL)	48.4	(42.0–54.9)**	26.1	(21.3–30.8)**
Mild (Hb 10 to <11 g/dL)	27.7	(23.0–32.3)§	18.5	(14.8–22.2)§
Moderate (Hb 7 to <10 g/dL)	20.4	(16.3–24.5)**	7.6	(5.0–10.1)**
Severe (Hb <7 g/dL)	0.31	(0–0.95)	0	—
**Nonpregnant women aged 15–49 yrs**				
Anemia††				
Any anemia (Hb <12 g/dL)	44.8	(38.5–51.0)**	31.1	(27.2–35.0)**
Mild (Hb 11 to <12 g/dL)	21.2	(16.7–25.7)	17.6	(14.6–20.7)
Moderate (Hb 8 to <11 g/dL)	22.5	(17.5–27.5)**	12.9	(10.7–15.2)**
Severe (Hb <8.0 g/dL)	1.0	(0–2.4)	0.5	(0–1.05)

**Abbreviations:** CI = confidence interval; WHZ = weight-for-height z-score; HAZ = height-for-age z-score; Hb = hemoglobin.

* Sample sizes: in Zaatari camp, 325; outside the camp, 479.

† Sample sizes: in Zaatari camp, 312; outside the camp, 467.

§ Statistically significant difference between in Zaatari camp and outside the camp, p<0.05.

¶ Sample sizes: in Zaatari camp, 318; outside the camp, 476.

** Statistically significant difference between in Zaatari camp and outside the camp, p<0.001.

†† Sample sizes: in Zaatari camp, 306; outside the camp, 618.

## Footnotes

* Definitions of anemia for children aged 6–59 months. Mild anemia: hemoglobin 10 to <11 g/dL; moderate anemia: hemoglobin 7 to <10 g/dL; severe anemia: hemoglobin <7 g/dL. Definitions of anemia for nonpregnant women aged 15–49 years. Mild anemia: hemoglobin 11 to <12 g/dL; moderate anemia: hemoglobin 8 to <11 g/dL; severe anemia: hemoglobin <8 g/dL.
